# Determinants of reach counts on medical social media: Evidence from a facebook community page metrics

**DOI:** 10.1002/jgf2.430

**Published:** 2021-03-05

**Authors:** Daisuke Nishioka, Toru Tsuboya

**Affiliations:** ^1^ Department of Medical Statistics, Research & Development Center Osaka Medical College Osaka Japan; ^2^ Japan Primary Care Association Commission on Social Determinants of Health (JPCA‐CSDH) Tokyo Japan; ^3^ Department of International and Community Oral Health Tohoku University Graduate School of Dentistry Miyagi Japan

**Keywords:** Facebook, *Mie‐statement*, social media, video

## Abstract

Sharing medical information using social media became popular. We aimed to identify what sort of posts can increase reach counts. We used metrics data of 40 posts on the Japan Primary Care Association Commission on Social Determinants of Health (JPCA‐CSDH) Facebook community page. The most popular post was the one about the position paper on health inequality (*Mie‐statement*). The video posts significantly earned 175% more reach counts (vs posts only with letters). Each one percent word counts increase was associated with a 31% increase in reach counts. Videos and more words may deliver medical information effectively.

## Graphical abstract

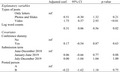

## 1 INTRODUCTION

Sharing medical information via social media has been attracting attention recently in medical education and public health.^1^, ^2^ Medical societies and medical care providers have shared information on social networking services (SNSs) (Facebook, Twitter, etc…), weblog communities, and blogs.^3^ Several studies have identified that visual articles including photographs or video can gain people's reach counts effectively in the marketing field.^4^, ^5^ However, the evidence of determinants of effective information delivery on social media is poorly documented and still inconclusive in the medical field,^6^ especially in Japan. Moreover, there are many restrictions on the collection of metrics data on social media, making it difficult to use the data for research.^7^ To close the gap, we analyzed the data of the Japan Primary Care Association Commission on Social Determinants of Health (JPCA‐CSDH) Facebook community page,^8^ whose metrics data were available with administrator authority of the community page. From the data, we aimed to identify what sort of posts can deliver the information more effectively.

## 2 MATERIALS AND METHODS

### 2.1 Data

The JPCA‐CSDH runs its Facebook page to share information on the activities and has 913 followers and two administrators who can post on the community, as of Dec. 15, 2020. For the present analysis, we analyzed data of Jun 2018 to December 2019. Facebook metrics data were obtained through the insight interface of Facebook community page functions. Each metrics data contained the date of posts; counts of reach, engagement, and reaction; contents of posts; and publishing statuses. We retrieved the data in January 2020. Because of a fixed available sample, the number of posts during the study period determined our sample size.^9^

### 2.2 Measurement and variables

#### 2.2.1 Outcome variables

We identified reach counts of each post from metrics data. Reach count was defined as “the number of unique people (not number of times) who saw a content” in Facebook, which we could use this variable as a number of people that information was delivered. Before we conduct the analyses, we log‐transformed reach counts to achieve normality.

#### 2.2.2 Explanatory variables

Based on previous studies on marketing researches,^3^, ^4^, ^5^ we extracted information of contents of posts from Facebook page and categorized posts into posts only with letters, photograph and slides posts, and video posts. Further, we counted the words of posts and log‐transformed the variable.

#### 2.2.3 Covariatess

Based on data availability, we used conference dummy variable that explains the posts about JPCA conferences and seminars (Yes/No), dummy variable of person who posted the content on the JPCA‐CSDH community page, to adjust for unmeasured characteristics of posts (A/B), and date of submission that was categorized into three terms; June to December 2018, January to June 2019, and July to December 2019, to adjust the effect of the number of followers on engagement.

### 2.3 Statistical analysis

First, we described the metrics and characteristics of overall posts. Second, we performed univariable linear regression analysis and calculated the crude coefficient of reach counts and its 95% confidence interval (CI) of each explanatory variable. Third, we performed multiple linear regression analysis to calculate the multivariable‐adjusted coefficient of each explanatory variable. There were no missing data for the variables. Analyses were performed by using STATA SE Ver.16.2 for these statistical analyses (Stata Corp.).

### 2.4 Ethical consideration

Our study did not include any personal information related to the posts and was thus exempted from ethical review.

## 3 RESULTS

The data included 40 posts from the administrators (A/B). The mean and standard deviation of reach counts was 1318 and 1190, and the maximum reach counts were 5600. The mean and standard deviation of word counts was 281.8 and 321.7. Among the 40 posts, 23 (57.5%) included photographs, and three (7.5%) included videos (Table 1). The post with the maximum 5600 reach counts was the post with a video where the JPCA position paper on health inequality (*Mie‐statement*) firstly delivered at the ninth‐annual conference in Mie in 2018. The univariable linear regression analysis showed that post with video was associated with higher counts of reach when compared with posts only with letters (Table S1). The result of multiple linear regression analyses showed that the adjusted coefficient of video posts was 1.75 (95% confidence interval [95%CI], 0.57‐2.93) when compared with posts only with letters. Besides, word count increase was associated with a higher reach counts (the adjusted coefficient 0.31, 95%CI: 0.06‐0.56) (Table 2).

**TABLE 1:** Summary of metrics and characteristics of each posts in Japan Primary Care Association Commission on Social Determinants of Health (JPCA‐CSDH) Facebook community page

	n of posts (N = 40)	% for N
Types of posts		
Only letters	14	35.0
Photographs and Slides	23	57.5
Video	3	7.5
Conference dummy		
No	12	30.0
Yes	28	70.0
Submission term		
June‐December 2018	13	32.5
January‐June 2019	19	47.5
July‐December 2019	8	20.0
Posted person		
A	36	90.0
B	4	10.0

	Reach counts
Mean	1318
Standard deviation	1190
Median	907
Minimum	140
Maximum	5600

	Word Counts
Mean	281.8
Standard deviation	321.7
Median	190
Minimum	8
Maximum	1558

**TABLE 2:** Adjusted coefficient and 95% confidence intervals for reach counts in the Japan Primary Care Association Commission on Social Determinants of Health (JPCA‐CSDH) Facebook community page: results of multivariable linear regression

	Adjusted coef.	95% CI		*P*‐value
Explanatory variables				
Types of posts				
Only letters	ref			
Photographs and Slides	0.51	−0.30	1.32	.21
Video	1.75	0.57	2.93	<.01
Log word counts	0.31	0.06	0.56	.02
Covariates				
Conference dummy				
No	ref			
Yes	0.17	−0.54	0.87	.63
Submission term				
June−December 2018	ref			
January−June 2019	0.06	−0.66	0.77	.88
July−December 2019	0.00	−1.04	1.04	1.00
Posted person				
A	ref			
B	−0.22	−1.62	1.18	.75

Abbreviations: CI, confidence interval; coef., Coefficient.

Multivariable linear regression analysis was adjusted by types of posts, log word counts, conference dummy variable, term of submissions, and posted person.

## 4 DISCUSSION

The post that earned the top access was the post from JPCA‐CSDH at the ninth‐annual conference delivered in video, and notably, the reach count was greater than the number of people who actually attended the meeting. The posts with video significantly earned 175% more reach counts when compared with posts only with letters. Each one percent word counts increase was associated with a 31% increase in reach counts. As far as we know, this is the first study that analyzed the data of social media in Japanese medical societies.

Our results of higher reach counts on video posts were consistent with results obtained in marketing fields. For example, Mariani et al identified that short video and the moderate long post has a positive impact on engagement by using Facebook data in Italy.^4^ Also, Ross reported that posts with photographs and videos were associated with more interactions globally.^5^

Our results implicated that social media enable more people or health professionals to access medical information. For example, members of a medical society who were unable to attend the annual congress or nonmembers of a medical society would become to be able to access medical information, which would result in improving the information gap, and making society better. Thus, medical societies could be accountable and increase their presence in the society. In the COVID‐19 era, remote congress and webinars become much more popular; thus, it is important and required to provide more information through video social media, to analyze the relevant factors in more detail, and to work on improving the quality of information dissemination.

There were several limitations to our study. First, because our analysis did not include the information of the time of submission, this may have biased our results. Engagement on the Facebook post was known to be different by the time of the submission.^4^, ^10^ Second, the generalizability of our results to other committees in JPCA and other medical societies is limited. Further, we analyzed data derived from Japanese Facebook community page and included counts of Japanese characters as word counts; the generalizability to other countries is unclear.

To conclude, our study suggested that the post with video or more words can disseminate medical information effectively through social media. Information dispatch using social media may narrow the information gap and may allow medical societies to fulfill their accountability and increase their presence in society. Further investigation with more samples and other broader metrics information would be required.

## CONFLICT OF INTEREST

The authors have stated explicitly that there are no conflicts of interest in connection with this article.

## Supporting information

Table S1
