# Supplementary material for: Determinants of reach counts on medical social media: Evidence from a facebook community page metrics
Source: J Gen Fam Med. 2021 Mar 5;22(5):300–3. doi: 10.1002/jgf2.430 (PMC8411400; doi:10.1002/jgf2.430)
Supplement: Supplementary file 1 — Table S1 [file JGF2-22-300-s001.docx]

**Supporting Information**

| Table S1. Crude coefficient and 95% confidence intervals for reach counts in the Japan Primary Care Association Commission on Social Determinants of Health (JPCA-CSDH) Facebook community page: results of univariable linear regression | | | | | |
| --- | --- | --- | --- | --- | --- |
|  |  | Crude coef. | 95% CI | | p-value |
| *Explanatory variables* | |  |  |  |  |
| Types of posts | |  |  |  |  |
|  | Only letters | ref |  |  |  |
|  | Photos and Slides | 0.24 | -0.37 | 0.84 | 0.43 |
|  | Video | 1.22 | 0.09 | 2.35 | 0.04 |
| Log word counts | |  |  |  |  |
|  |  | 0.18 | -0.03 | 0.39 | 0.10 |
| *Covariates* | |  |  |  |  |
| Conference dummy | |  |  |  |  |
|  | No | ref |  |  |  |
|  | Yes | 0.30 | -0.33 | 0.93 | 0.35 |
| Submission term | |  |  |  |  |
|  | June-December 2018 | ref |  |  |  |
|  | January-June 2019 | 0.13 | -0.54 | 0.81 | 0.69 |
|  | July-December 2019 | 0.14 | -0.71 | 0.98 | 0.74 |
| Posted person | |  |  |  |  |
|  | A | ref |  |  |  |
|  | B | 0.03 | -0.94 | 1.01 | 0.94 |

CI, confidence interval; coef., Coefficient.
